# Mechanical Reinforcement of Diopside Bone Scaffolds with Carbon Nanotubes

**DOI:** 10.3390/ijms151019319

**Published:** 2014-10-23

**Authors:** Cijun Shuai, Tingting Liu, Chengde Gao, Pei Feng, Shuping Peng

**Affiliations:** 1 Shenzhen Research Institute, Central South University, Shenzhen 518057, China; E-Mail: shuai@csu.edu.cn; 2 State Key Laboratory of High Performance Complex Manufacturing, Central South University, Changsha 410083, China; E-Mails: liutingting@csu.edu.cn (T.L.); gaochengde@csu.edu.cn (C.G.); fengpei@csu.edu.cn (P.F.); 3 Cancer Research Institute, Central South University, Changsha 410078, China

**Keywords:** carbon nanotubes, diopside, mechanical reinforcement, scaffolds

## Abstract

Carbon nanotubes are ideal candidates for the mechanical reinforcement of ceramic due to their excellent mechanical properties, high aspect ratio and nanometer scale diameter. In this study, the effects of multi-walled carbon nanotubes (MWCNTs) on the mechanical properties of diopside (Di) scaffolds fabricated by selective laser sintering were investigated. Results showed that compressive strength and fracture toughness improved significantly with increasing MWCNTs from 0.5 to 2 wt %, and then declined with increasing MWCNTs to 5 wt %. Compressive strength and fracture toughness were enhanced by 106% and 21%, respectively. The reinforcing mechanisms were identified as crack deflection, MWCNTs crack bridging and pull-out. Further, the scaffolds exhibited good apatite-formation ability and supported adhesion and proliferation of cells *in vitro*.

## 1. Introduction

As an interdisciplinary field, bone tissue engineering applies the principles and methods of biology and engineering to the development of substitutes for damaged bone tissue [1,2,3,4]. In general, biocompatible scaffolds, which act as a temporary mechanical support for cell attachment, propagation and differentiation, are an essential component for bone tissue engineering [5,6,7,8]; furthermore, their architecture determines the final shape of the new bone. Therefore, the scaffolds must have the desired external shape, defined internal structure, high porosity and sufficient biomechanical support [9,10]. Selective laser sintering (SLS) is advantageous for fabricating the scaffolds since it allows construction of scaffolds with accurate geometries and controlled internal structure. In SLS processing, scaffolds are built by sequentially fusing powders on a powder bed, layer by layer [11,12].

Diopside (CaMgSi_2_O_6_, Di) belongs to the CaO–MgO–SiO_2_ system, which has the ability to release Mg and Si ions. Mg ions are mainly responsible for the apatite-formation ability of the scaffolds, and Si ions are able to stimulate cell growth and differentiation on the scaffolds [13,14]. Therefore, Di is considered to be an attractive material for bone tissue engineering applications. Unfortunately, low compressive strength and fracture toughness of Di limit its use for low-strength parts, which leads to serious obstacles for wider applications.

Carbon nanotubes (CNTs) are allotropic variants of carbon with cylindrical nanostructures that exhibit superior flexibility, excellent stiffness and high Young’s modulus [15]. Therefore, CNTs are considered to be potential candidates for ceramic reinforcement. Recently, a number of studies have focused on enhancing the mechanical properties with CNTs. MA *et al.* [16] prepared CNTs/SiC ceramic using the hot-press method and found that the mechanical properties of the SiC ceramic increased by the addition of CNTs. Ning *et al.* [17] fabricated CNT-SiO_2_ composites by hot pressure sintering. It was concluded that the bending strength and fracture toughness of the 5vol. % CNT-SiO_2_ composite were enhanced 65% and 100% compared with the monolithic SiO_2_. In addition, CNTs can support the adhesion and growth of osteoblasts, myoblasts and neurons, which offer a wide range of opportunities for bone tissue engineering applications [18,19,20,21,22].

In this study, porous Di scaffolds reinforced with multi-walled carbon nanotubes (MWCNTs) were prepared by SLS. The microstructure was examined with scanning electron microscope (SEM) and Raman spectroscopy. Moreover, mechanical tests were performed to investigate the effect of MWCNTs on the compressive strength and fracture toughness of Di scaffolds. In addition, bioactivity and biocompatibility were evaluated by immersing the scaffolds in simulated body fluid (SBF) and culturing with MG63 cells, respectively.

## 2. Results and Discussion

### 2.1. Scaffold Characterizations

**Figure 1:**
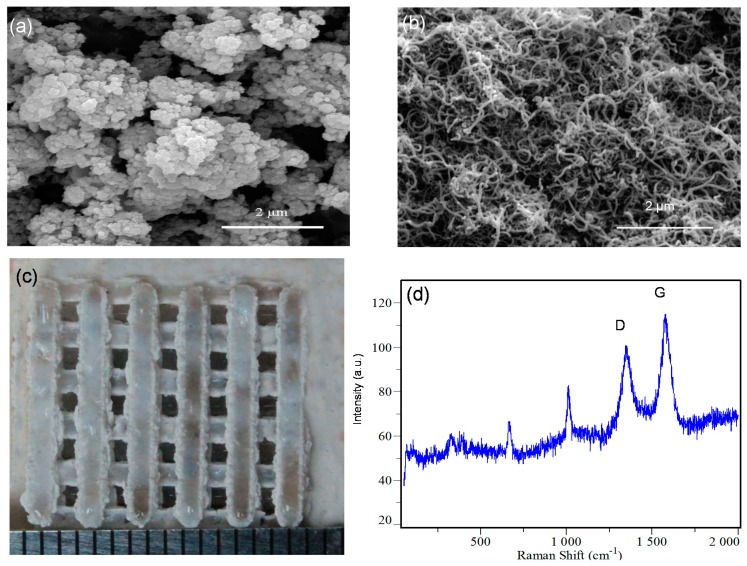
Morphology of the raw powders (**a**) Di powders; and (**b**) MWCNTs powders; (**c**) Di scaffold prepared by SLS; (**d**) Raman spectrum of the prepared scaffold, D: D band of CNTs; G: G band of CNTs.

The morphologies of raw Di and MWCNTs are shown in Figure 1a,b, respectively. It was observed that Di particles approached a spherical shape, and most of the MWCNTs were bound together. The average diameter of Di and MWCNTs were about 0.2 μm and 30 nm, respectively.

The Di scaffold reinforced with MWCNTs is shown in Figure 1c. It had interconnected porous structure which was crucial for transportation of nutrients and the removal of waste. The scaffold was 16 mm in length and 16 mm in width. The strut size was 1.5 mm and the pore size was 1.5 mm. Raman spectrum of the scaffold was shown in Figure 1d. It exhibited distinct peaks at a Raman shift of 1340–1360 and 1570–1580 cm^−1^, corresponding to the characteristic D band and G band of CNTs [23], respectively, which indicated that MWCNTs existed in the scaffold after sintering.

### 2.2. Mechanical Properties

**Figure 2:**
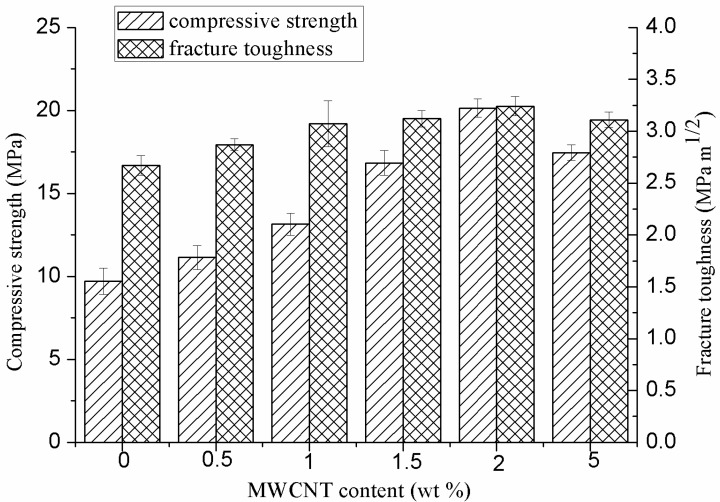
Compressive strength and fracture toughness of the scaffolds with different MWCNTs content.

The effects of MWCNTs on the compressive strength and fracture toughness of the scaffolds are shown in Figure 2. The addition of MWCNTs resulted in an increase in compressive strength and fracture toughness. The compressive strength increased dramatically with the increase of MWCNTs content up to 2 wt %, and then decreased with further increasing MWCNTs content to 5 wt %. The fracture toughness of the scaffold showed a similar trend as the compressive strength. The optimum compressive strength and fracture toughness reached 20.1 ± 0.6 MPa and 3.2 ± 0.1 MPa·m^1/2^, when 2 wt % MWCNTs were introduced. The compressive strength and fracture toughness of the scaffold with 2 wt % MWCNTs were enhanced by 106% and 21% compared to the scaffold without MWCNTs.

**Figure 3:**
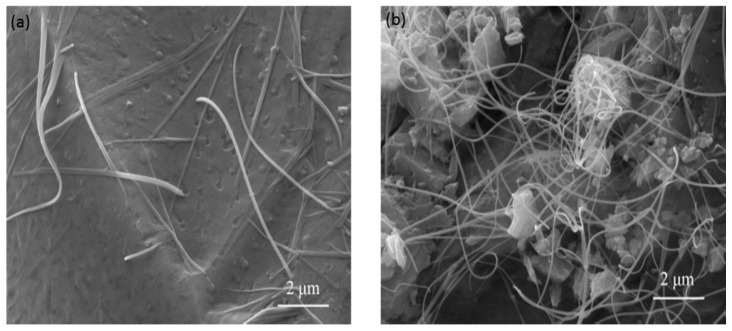
The surface morphology of (**a**) Di scaffold with 2 wt % MWCNTs; and (**b**) Di scaffold with 5 wt % MWCNTs.

The surface morphologies of Di scaffold with 2 and 5 wt % MWCNTs are shown in Figure 3. It was observed that when 2 wt % MWCNTs were incorporated, MWCNTs uniformly distributed in the scaffold (Figure 3a). Homogeneous dispersion of MWCNTs could enhance their pulling-out strength and thus increase the strength of the scaffold. However, further increasing the MWCNTs to 5 wt %, it was observed that MWCNTs agglomerated in the scaffold (Figure 3b). Agglomeration of MWCNTs in the scaffold not only made the strength of the scaffold decline, but also resulted in formation of defects. Consequently, the dispersing properties of MWCNTs were crucial to their reinforcing ability.

### 2.3. Discussion on Reinforcing Mechanism

**Figure 4:**
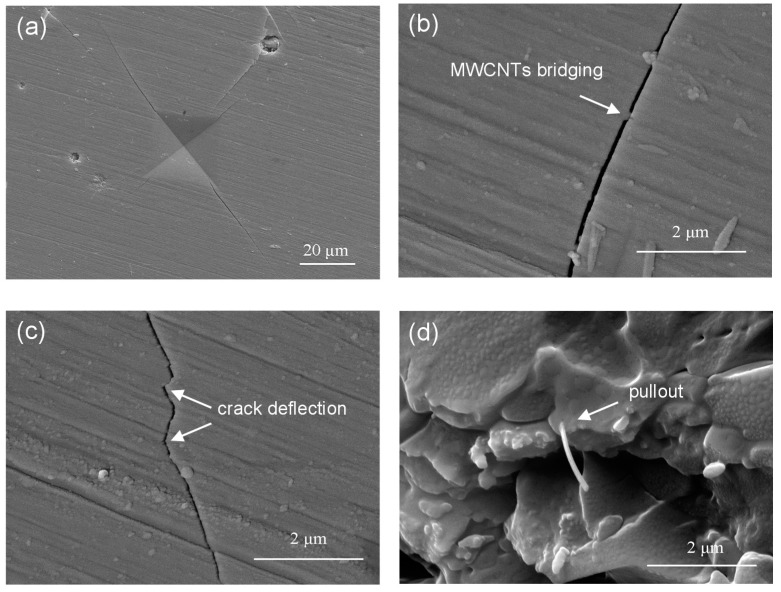
SEM of (**a**) indentation crack propagation of Di scaffold with 2 wt % MWCNTs; (**b**) MWCNTs crack bridging; (**c**) crack deflection; (**d**) MWCNTs pull-out at the fracture surface of the scaffold.

The crack propagation path was obtained on the Di scaffold with 2 wt % MWCNTs using microhardness indentation at a load of 4.9 N (Figure 4a). During the fracture process of the scaffold, it mainly presented three kinds of reinforcing behaviors such as crack deflection, MWCNTs crack bridging and pull-out. For bridging effect of MWCNTs, MWCNTs connected two sides of the crack before fracturing and thus restrained further crack propagation (Figure 4b). The second toughening mechanism was crack deflection. The propagating crack changed direction as it encountered MWCNTs (Figure 4c). The effect of MWCNTs bridging and crack deflection was beneficial for forming a new energy-absorption mechanism and consequently enhanced strength and toughness of the scaffolds. MWCNTs pull-out was another important toughening mechanism. It was showed that MWCNTs were embedded in the scaffold, which was wrapped tightly by the Di matrix (Figure 4d). MWCNTs pull-out at the fracture surface was efficient in transferring the load from the Di matrix to the MWCNTs, leading to the significant improvement of toughness.

### 2.4. Bioactivity Evaluation

**Figure 5:**
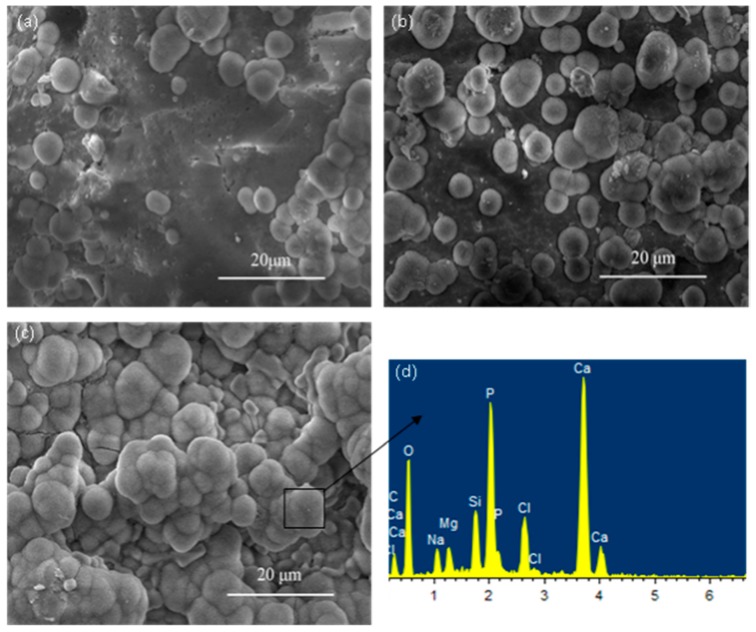
Bone-like apatite precipitated on the Di scaffolds for different periods of immersion (**a**) 7 days; (**b**) 14 days; (**c**) 21 days; and EDS spectra of (**d**) the globular bone-like apatite in (**c**).

The bioactivity of the scaffold was evaluated by determining the changes in surface morphology and bone-like apatite layer growth after soaking in SBF. After immersion for 7 days, the surface of the scaffold was covered with some agglomerates of globular particles (Figure 5a). When the immersion time increased to 14 days, the amount of the globular particles grew rapidly (Figure 5b). As the soaking time increased to 21 days, the globular particles formed a dense and continuous layer on the surface of the scaffold (Figure 5c). EDS analysis showed that particles were mainly composed of Calcium and Phosphorus element, and the Ca/P ratio correspond to the hydroxyapatite ratio, which could be indicative of the forming of apatite layer. The forming of apatite layer on biomaterials is supposed to be the precondition for inducing bone formation.

### 2.5. In Vitro Cellular Behavior

**Figure 6:**
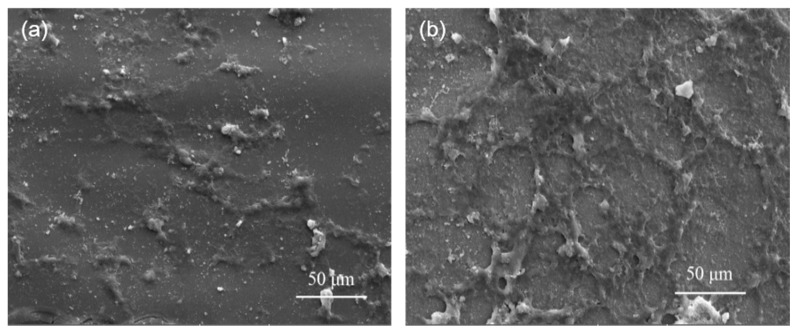
MG-63 cells cultured on Di scaffolds for (**a**) 3 days; and (**b**) 7 days.

The SEM micrographs of Di scaffold cultured with MG-63 cells for 3 and 7 days were shown in Figure 6. It was obvious that a small number of elongated and fusiform cells attached on the scaffold and presented a close contact with the scaffold after 3 days of culture (Figure 6a). When the culture time increased to 7 days, significant spreading of cells with lamellipodia was observed on the surface of the scaffold (Figure 6b), which indicated good cellular attachment and migration. In addition, the extracellular matrix (ECM) that was secreted by the cells was observed on the scaffold, which was crucial for cell phenotype, cell migration, and gene expression [24]. The results suggested that the developed Di scaffold provided favourable conditions for adhesion, spread and proliferation of cells.

## 3. Experimental Section

### 3.1. Materials and Fabrication 

The Di powder was supplied by Kun Shan Chinese Technology New Materials Co. Ltd. (Kunshan, China). MWCNTs supplied by Nanjing XFNano Material Tech Co., Ltd. (Nanjing, China). were used as reinforcement in this study. Since MWCNTs usually agglomerate and entangle together due to Van der Waals force, and are extremely difficult to disperse in the matrix [25], the key issue in the development of MWCNTs-reinforced scaffolds is to obtain a uniform dispersion of MWCNTs in the matrix. In the experiment, Di and different weight percentages of MWCNTs (0.5, 1, 1.5, 2 and 5 wt %) powders were dispersed in alcohol, respectively, and then mixed in an ultrasonic shaker for 30 min. Subsequently, the mixed powders were degassed in a vacuum oven to evaporate alcohol for 2 h, followed by mechanically stirring in a mortar for 30 min.

Di scaffolds were fabricated using a self-developed SLS system which was introduced in detail in the previous papers [26,27]. In brief, the SLS system consisted of a laser sintering system, an optical focusing system, a control system and a three-dimensional motion platform. The laser sintering machine is equipped with a 100 W CO_2_ laser (Firestar^®^ t-Series, Synrad Co., WA, USA). The energy intensity across the laser beam diameter follows a Gaussian distribution. The composite powders were sintered at the following process parameters: laser spot diameter was 2 mm, laser power was 6 W and scanning speed was 100 mm/min, respectively. When SLS processing was completed, the scaffolds were cooled back to room temperature. Excess powders surrounding the scaffolds were brushed off and the scaffolds were cleaned by blowing compressed air.

### 3.2. Structural Characterizations

Morphologies of the scaffolds were observed by SEM (TESCAN MIRA3 LMU, Brno, Czech Republic) with a working acceleration voltage of 20 KV. All the specimens were pre-coated with gold/palladium under an argon atmosphere.

A LabRAM HR800 spectrometer (HORIBA Jobin Yvon, Lille, France) was used to verify the presence of MWCNTs in the scaffolds. Readings were collected with varying data accumulations with the use of a laser power of 0.8 mW and a laser excitation of 532 nm. Defect induced D-band and graphite-like G-band were observed and compared qualitatively.

### 3.3. Mechanical Measurements

The compressive strengths of the scaffolds were tested by a universal testing machine (Shanghai Zhuoji Instruments Co. Ltd., Shanghai, China). All scaffolds were compressed at a cross head speed of 0.5 mm/min until the scaffolds were crushed completely. Fracture toughness of the scaffolds was measured using a Vickers Microindenter (Digital Micro Hardness Tester, HXD-1000TM/LCD, Shanghai Taiming Optical Instrument Co. Ltd., Shanghai, China) with a load of 4.9 N and a hold of 15 s. The scaffolds were mounted in epoxy vertically, polished and subjected to indentation on the surfaces. Five samples for each group were used to obtain the average value along with their standard deviation. The fracture toughness was evaluated with Equation (1) [28]:

*K*_IC_ = 0.0824(*P*/*C*^1.5^) (1)

where *K*_IC_ was the fracture toughness, *P* was the applied indenter load, and *C* was the crack length.

### 3.4. Bioactivity and Cytocompatibility 

The bioactivity of the scaffolds was evaluated by studying the growing process of the apatite layer on the surface of the scaffolds after being immersed in simulated body fluid (SBF), which has the same ions and ion concentration as that of body fluid. The synthesizing process of SBF was described by Kokubo and Cho [29]. The scaffolds were soaked in SBF at 37 °C for 7, 14 and 21 days, then retrieved from the SBF, washed with distilled water and dried in a vacuum drying oven for characterization of apatite mineralization by SEM. 

Human osteosarcoma MG-63 cells from American Type Culture (ATCC, Rockville, MD, USA) were used in this research to evaluate the biocompatibility of the scaffolds. MG-63 cells were cultured in Dulbecco’s modified Eagle’s medium (DMEM) supplemented with 10% fetal calf serum, 2 mM glutamine and 100 μg/mL penicillin–streptomycin at 37 °C. The scaffolds were sterilized with an ethanol series (50%, 70%, 90%) before being washed with phosphate-buffered saline (PBS) for 24 h. The cells at a density of 4 × 10^5^ cells/cm^2^ were seeded onto the scaffolds placed in a 24-well plate. Subsequently, the scaffolds were maintained in the incubator for 3 and 7 days. After being washed with PBS, the scaffolds were dehydrated using graded ethanol changes, critical point dried, gold coated under an argon atmosphere, and examined by SEM.

## 4. Conclusions 

Di scaffolds with porous architecture were prepared by SLS. The mechanical test results demonstrated that compressive strength and fracture toughness increased with increasing MWCNTs content up to 2 wt %. The enhancement of mechanical properties was ascribed to crack deflection, MWCNTs crack bridging and pull-out. However, further additions of MWCNTs negatively affected the mechanical properties due to the agglomeration of MWCNTs. Further, the scaffolds presented excellent apatite-formation ability in SBF and enabled adhesion, proliferation, and differentiation of the MG63 cells. Thus, it is proposed that MWCNT-reinforced Di scaffolds have potential for use in bone tissue engineering.
